# Natural Anti-Inflammatory Products/Compounds: Hopes and Reality

**DOI:** 10.1155/2015/374239

**Published:** 2015-11-29

**Authors:** Barbara Romano, Asif Jilani Iqbal, Francesco Maione

**Affiliations:** ^1^Department of Pharmacy, University of Naples Federico II, Via Domenico Montesano 49, 80131 Naples, Italy; ^2^Sir William Dunn School of Pathology, University of Oxford, South Parks Road, Oxford OX1 3RE, UK

Inflammation is a complex biological response to injury as a result of different* stimuli* such as pathogens, damaged cells, or irritants. Nowadays, the commercially approved anti-inflammatory drugs are represented by nonsteroidal anti-inflammatory drugs (NSAID), glucocorticoids (SAID), and in some cases immunosuppressant and/or biological drugs. These agents are effective for the relief of the main inflammatory symptoms. However, they induce severe side effects, whereas most of them are inadequate for chronic use.

Starting from these premises, the demand of new effective and safely anti-inflammatory drugs has furthered research into new therapeutic approaches. The recent and emerging scientific community slant is oriented to the herbal medicines that could represent a treasure for the discovery of new active compounds and for the development of new drugs and potentially useful therapeutic agents.

The articles contained in this special issue include both reviews and basic scientific studies focused on characterizing the molecular mechanisms of the inflammatory process in humans and also in animal models.

In particular, on this special issue the attention on the role of proinflammatory mediators and pathways such as IL-1, IL-6, mPGES-1, and NF-B had been pointed out (“*Moringa oleifera* Flower Extract Suppresses the Activation of Inflammatory Mediators in Lipopolysaccharide-Stimulated RAW 264.7 Macrophages* via* NF-*κ*B Pathway” by W. S. Tan et al. and “Downregulation of mPGES-1 Expression* via* EGR1 Plays an Important Role in Inhibition of Caffeine on PGE_2_ Synthesis of HBx(+) Hepatocytes” by Y. Ma et al. and “Anti-Inflammatory Effect of 1,3,5,7-Tetrahydroxy-8-isoprenylxanthone Isolated from Twigs of* Garcinia esculenta* on Stimulated Macrophage” by D.-D. Zhang et al.).

Moreover, this special issue has highlighted the importance of some mechanism and strategy involved in inflammatory bowel disease and experimental model of osteoarthritis (“Botanical Drugs as an Emerging Strategy in Inflammatory Bowel Disease: A Review” by F. Algieri et al. and “Therapeutic Effect of Chenodeoxycholic Acid in an Experimental Rabbit Model of Osteoarthritis” by Z. Yan et al.). Finally, interesting contributions regarding the anti-inflammatory activity of marine diterpenoids and leishmaniasis inflammatory response have been successfully submitted to this special issue (“Marine Diterpenoids as Potential Anti-Inflammatory Agents” by Y. González et al. and “Natural Products: Insights into Leishmaniasis Inflammatory Response” by I. A. Rodrigues et al.).

We hope that this special issue will stimulate the interest of the scientific community involved in studying the effects of natural products/compounds on different fields of interests such as inflammation and pain. The papers published here will surely contribute to proposing new additional insights into the mechanism of several conditions as well as to suggesting new diagnostic alternatives and therapeutic targets in widespread pathologies. The discovery of the new is, as always, anchored to the recourse of the old.

